# Supplementary material for: A Critical View of the Application of the APEX Software (Aqueous Photochemistry of Environmentally-Occurring Xenobiotics) to Predict Photoreaction Kinetics in Surface Freshwaters
Source: Molecules. 2019 Dec 18;25(1):9. doi: 10.3390/molecules25010009 (PMC7017383; doi:10.3390/molecules25010009)
Supplement: Supplementary file 1 [file molecules-25-00009-s001.zip › 3.2.4_gcc-4.4.0.zip!/3.2.4_gcc-4.4.0/3.2.4_gcc-4.4.0/MSYS/doc/msys/MSYS_WELCOME.rtf]

MSYS - Minimal SYStem
Version 1.0.11
http://www.mingw.org/wiki/msys/

Welcome to the world of MSYS and MinGW.  Minimal SYStem is a minimal POSIX system used in the Win32 OS to accomplish configuration and making of packages.

MSYS is a fork of Cygwin and is more friendly to the Win32 user.  It offers only a user land environment for MinGW development.  It converts posix paths before executing a "native" win32 program.  This allows the users of the MinGW version of GCC to port and build packages in a GNU familiar way and removes the UNIX complexities that Cygwin adds.

MSYS has it's own mailing list, mailto:mingw-msys@lists.sf.net, so if you have questions or experience problems ask on that list please.  I have many plans for MSYS and MinGW and plan for a new release of not more than a six weeks span.  Announcements of the new release will be done in both the news posts on the http://www.mingw.org web site and the mingw-msys mail list. 

More documentation can be found in the /doc/msys directory.  Please be sure to read the documents in that directory.

Enjoy,
Earnie
Earnie@users.sf.net
